# The SARS-CoV-2 Pandemic in High Income Countries Such as Canada: A Better Way Forward Without Lockdowns

**DOI:** 10.3389/fpubh.2021.715904

**Published:** 2021-11-22

**Authors:** Ari R. Joffe, David Redman

**Affiliations:** ^1^Department of Pediatrics and John Dossetor Health Ethics Center, Faculty of Medicine, University of Alberta, Edmonton, AB, Canada; ^2^Retired LCol, Alberta Emergency Management Agency, St. Paul, AB, Canada

**Keywords:** COVID-19, emergency management (EM), lockdowns, pandemic, response

## Abstract

The SARS-CoV-2 pandemic has caused tragic morbidity and mortality. In attempt to reduce this morbidity and mortality, most countries implemented population-wide lockdowns. Here we show that the lockdowns were based on several flawed assumptions, including “no one is protected until everyone is protected,” “lockdowns are highly effective to reduce transmission,” “lockdowns have a favorable cost-benefit balance,” and “lockdowns are the only effective option.” Focusing on the latter, we discuss that Emergency Management principles provide a better way forward to manage the public emergency of the pandemic. Specifically, there are three priorities including the following: first, protect those most at risk by separating them from the threat (mitigation); second, ensure critical infrastructure is ready for people who get sick (preparation and response); and third, shift the response from fear to confidence (recovery). We argue that, based on Emergency Management principles, the age-dependent risk from SARS-CoV-2, the minimal (at best) efficacy of lockdowns, and the terrible cost-benefit trade-offs of lockdowns, we need to reset the pandemic response. We can manage risk and save more lives from both COVID-19 and lockdowns, thus achieving far better outcomes in both the short- and long-term.

## Introduction

The Severe Acute Respiratory Syndrome Coronavirus-2 (SARS-CoV-2) that causes Coronavirus Disease 2019 (COVID-19) was declared a pandemic in March 2020. Governments have made (often suboptimal) decisions to manage the pandemic crisis, often focusing only on the many cases and deaths worldwide caused by COVID-19. Attempting to “flatten the curve” of cases and deaths, governments have implemented unprecedented restrictions on Charter rights and freedoms, the lockdowns and “circuit-breakers” (time-limited lockdowns that were not time-limited). By lockdown we mean any combination of non-pharmaceutical interventions (NPIs) that imposed mandated social distancing measures and/or restrictions on mobility (e.g., school closures, restaurants and large shopping centers closure, workplace closures, limits on gathering sizes, closure of public transport, stay-at-home orders, and even curfews). Many studies we will refer to used a stringency index to indicate the severity of these lockdown measures; as stringency becomes more severe, the collateral damage may become worse. We present several mistaken assumptions that we believe have led to the lockdown approach to the pandemic. We argue that a better response to the pandemic must occur, one that saves more lives, causes fewer harms, and restores trust.

## Mistaken Assumption: No One is Protected Until Everyone is Protected

Many more infections occur than are detected and diagnosed as cases. The *case* fatality rate is thus much higher than the *infection* fatality rate (IFR, the number of people with SARS-CoV-2 infection that will die of COVID-19), often over 10-times higher (1, 2). In an analysis of international reports the median IFR was 0.23%, median for people <70 years was 0.05% (99.95% infection survival) (1), and this was lower than for influenza in people <50 years (Table 1). Other reviews also found that IFR increased with age, with an inflection point at approximately age 70 years (2, 4). A review of IFR studies suggested an overall population IFR of 0.15% (3, 5). The case fatality rate in Canada is compatible with these data (Table 2).

**Table 1:** SARS-CoV-2 age-specific *infection* fatality rates compared to infection fatality rates from Influenza in the United States.

	**Infection fatality rate for SARS-CoV-2 in systematic reviews^a^**			**Infection fatality rate for influenza**
**Age group**	**Ioannidis (1, 3) Median% (range)**	**O'Driscoll et al. (4) Median% (95% CrI)**	**Levin et al. (2) Mean (95% CI)**	**USA 2018–19 (assuming 20% asymptomatic)^b^**
Age ≤ 70 y	0.05% (0.00, 0.31)	-	-	Age <65 years: 0.021
Age 0–4 y	0.0027	0.003 (0.002, 0.003)	0.001 (0.0007, 0.0013)	0.0059
Age 5–9 y		0.001 (0.000, 0.001)		0.0022
Age 10–14 y		0.001 (0.001, 0.001)	0.003 (0.002, 0.004)	
Age 15–19 y		0.003 (0.002, 0.003)		
Age 20–24 y	0.014	0.006 (0.005, 0.008)	0.011 (0.009, 0.013)	0.016
Age 25–29 y		0.013 (0.011, 0.015)		
Age 30–34 y	0.031	0.024 (0.021, 0.028)	0.037 (0.031, 0.043)	
Age 35–39 y		0.040 (0.034, 0.047)		
Age 40–44 y	0.082	0.075 (0.064, 0.087)	0.123 (0.108, 0.141)	
Age 45–49 y		0.121 (0.104, 0.140)		
Age 50–54 y	0.27	0.207 (0.177, 0.239)	0.413 (0.362, 0.471)	0.049
Age 55–59 y		0.323 (0.277, 0.373)		
Age 60–64 y	0.59	0.456 (0.392, 0.527)	1.38 (1.19, 1.61)	
Age 65–69 y		1.075 (0.921, 1.244)		0.67
Age 70–74 y	2.4 (0.3, 7.2)	1.674 (1.435, 1.937)	4.62 (3.83, 5.57)	
Age 75–79 y		3.203 (2.744, 3.705)		
Age 80+ y		8.292 (7.105, 9.593)	15.46 (12.2, 19.5)	

a *Ioannidis (1) found median population IFR 0.23% (Range 0.00, 1.54); median IFR for countries with population mortality lower than the global average, higher than the global average with <500 deaths/million, and higher than global average with >500 deaths/million were 0.09, 0.20, and 0.57%. The age group data is from Axfors & Ioannidis (3), and in age 70+ reflects community dwelling people. O'Driscoll et al. (4) excluded deaths in nursing homes from their IFR modeling; the IFR estimated in nursing homes was 22.25% (95% CrI 19.06, 25.74). Levin et al. (2) used mean (95% CI) in their metaregression for IFR despite the I^2^ (heterogeneity) of 97.0; median is more appropriate when there is such high heterogeneity (I,4). For example, in Levin et al. (2) the median IFR for age 55–64 years (based on their Figure) was 0.37%*.

b *Influenza data from: https://www.cdc.gov/flu/about/burden/2018-2019.html?web=1&wdLOR=c444B9526-9E67-42D6-81A4-055CE0C2DF1A*.

**Table 2:** *Case* hospitalization, intensive care unit, and fatality rate for SARS-CoV-2 in Canada and selected Provinces as of mid-May, 2021^a^.

**Case age group**	**Case hospitalization rate (%)**		**Case ICU admission rate (%)**		**Case fatality rate (%)**					
	**Canada**	**Alberta**	**Canada**	**Alberta**	**Canada**		**Alberta**		**Ontario**	
	**May 14**	**May 15**	**May 14**	**May 15**	**March 2**	**May 14**	**March 2**	**May 15**	**March 2**	**May 17**
Age ≤ 70	3.0	2.7	0.7	0.6	0.33	0.29	0.24	0.19	0.34	0.30
Age 0–4 y	0.5	1.0	0.06	0.2	0.003	0.005	0	0	0.005	0.005
Age 5–9 y		0.2		0.1			0	0		
Age 10–19 y		0.5		0.06			0	0		
Age 20–29 y	1.0	1.2	0.1	0.1	0.02	0.02	0.04	0.02	0.02	0.02
Age 30–39 y	2.0	1.9	0.3	0.2	0.05	0.05	0.04	0.03	0.04	0.05
Age 40–49 y	3.0	3.0	0.6	0.6	0.13	0.13	0.12	0.11	0.14	0.13
Age 50–59 y	5.5	5.8	1.4	1.4	0.50	0.46	0.39	0.33	0.51	0.49
Age 60–69 y	11.1	10.4	3.0	2.9	2.29	2.02	1.93	1.64	2.13	1.92
Age 70–79 y	23.7	22.3	5.2	4.4	9.85	8.65	7.65	6.86	8.70	7.41
Age 80y+	27.2	28.5	1.9	1.3	24.87	23.9	22.29	21.46	23.51	22.11

*Despite steadily increasing proportions of “variants of concern,” the CFR in all age groups in May 2021 is the same or lower than in March 2021. Of note, in Alberta as of May 18 2021 the CFR in VOC (94% of which were B.1.1.7) = 148/42,108 = 0.35% compared to overall CFR = 2,158/22,1467 = 0.97%. The number of infections is about 5-10X more than the identified cases: to convert case rates in the table to infection rates divide by at least 5 (or, more likely, by 10) (2, 5)*.

a *Canadian data: https://health-infobase.canada.ca/covid-19/epidemiological-summary-covid-19-cases.html (6), the Case Fatality Rate in age 20–49 yr is 0.062% on March 2 and 0.063% on May 14, suggesting the Infection Fatality Rate [which is usually at least 5 times lower] is <0.013%; Alberta data: https://www.alberta.ca/stats/covid-19-alberta-statistics.htm#severe-outcomes (7); Ontario data: https://covid-19.ontario.ca/data*.

People ≥70 years, and 60–69 years with multiple severe comorbidities (e.g., obesity, diabetes mellites, kidney-disease, dementia) are at most risk for severe outcomes from SARS-CoV-2 (6, 8). In Canada, as of March 2, 89% of deaths occurred in people ≥70 years, and most (68%) other deaths were in people 60–69 years, usually with multiple comorbidities (7, 9). Of deaths, >70% occurred in long-term care homes for older people (10). People ≥70 years accounted for many hospitalizations (55%), intensive-care admissions (39%), and deaths (89%) across Canada (7). These are the groups of people that required protection.

Another consistent association with hospitalization and mortality from COVID-19 is social vulnerability, including risks from poverty or low-income (often associated with essential high-exposure occupations without job security, unemployment, lower education attainment, lack of health insurance, and food insecurity), household composition (e.g., single parent home, people with disability) and type (e.g., household crowding, lack of a vehicle, use of public transport for work commuting), and racial or ethnic minority status (e.g., African American, American Indian, Hispanic, often with limited English-speaking ability) (11, 12). People from racial and ethnic minorities have been more adversely affected by the pandemic, and not due to inherent biologic differences. Minority groups are a proxy for the “structural racism and social inequalities [affecting their social determinants of health] embedded within the economic, political, health care, criminal justice, and other systems and social structures (13)” (13–18). These result in higher exposure to and onward transmission of SARS-CoV-2 (e.g., inability to socially distance, work from home, or even wash hands; multi-generational housing units; densely populated communities), higher susceptibility to severe COVID-19 (e.g., more chronic non-communicable diseases, poor health due to stress and food insecurity, poor access to communication, poor access to healthcare), and lack of timely access to quality healthcare once sick (11–21). Within these vulnerable groups, people who are older and people with multiple severe comorbidities require specific strategies for focused protection.

The absolute number of cases and deaths we are bombarded with daily are given without denominators nor context. In Canada, in non-pandemic 2019, there were >797 deaths/day and 291,000 deaths/yr (22). Over the past 12 months, as of March 2, 2021, there have been 21,799 COVID-19-associated deaths in Canada, thus accounting for <7% of overall deaths (7). Many of these deaths were in people who in other years may have died from other causes. Globally, over the first year of the pandemic, COVID-19 accounted for 4.1% of deaths (23, 24); motor vehicle collisions, alcohol use, tobacco use, fossil fuel combustion fine particulate matter pollution, and poor diet accounted for 2.3, 5.1, 13.7, 14.9, and 18.8% of deaths respectively (25–29).

A focus on case counts is not as important as the data about hospitalization, intensive care admission, and mortality, for several reasons: the large majority of cases (milder symptomatic cases and asymptomatic infections) are not detected because they are not tested (1, 2), lockdowns were intended to preserve hospital and intensive care healthcare capacity and prevent deaths, and the risk to everyone (anyone, of any age or demographic) is not so high that protection of *all* from all cases must be a goal. For example, population-wide lockdowns are not used for seasonal influenza that has similar risk as COVID-19 to those aged <50 years old. We do not close down society in attempt to protect everyone from highly prevalent causes of death including motor vehicle collisions, alcohol use, tobacco use, fossil fuel combustion, and poor diet. Instead, those people at reasonably high risk for adverse outcomes from these threats are the people who require focused protection. For COVID-19, this includes people aged ≥70 years, and people aged 60–69 years with multiple severe comorbidities. The assumption that everyone can be protected equally, when their risks are markedly different, only contributed to population fear.

## Mistaken Assumption: Lockdowns are Highly Effective to Reduce Transmission

Many assume there is good evidence that lockdowns are required to protect high-risk people from SARS-CoV-2 infection. The efficacy of lockdowns to reduce transmission, cases and deaths has likely, at best, been highly exaggerated. Many studies found that more restrictive lockdowns in different international locations were not associated with any clear effect on “flattening-the-curve” of cases or deaths (Tables 3, 4) (30–61). Unrecognized confounding (e.g., seasonality, population density and age-structure, life-expectancy progression, etc.) and modeling errors likely account for studies that have suggested lockdowns to be an effective strategy (31, 46, 51, 58). It is possible that lockdowns cannot markedly reduce transmission as people continue to carry out their essential activities, and as, in the attempt to avert infections inside a system, lockdown restrictions in fact create forced congestion (and hence transmission) in other parts of the system (62). For example, young people may have returned to or stayed home (where most transmission occurs) with older parents due to unemployment or university closures, and risk may have simply been shifted from the professional class to the working class, who cannot afford not to work (63, 64). The incremental effect of lockdowns on transmission to those at high-risk may be nonexistent after accounting for endogenous behavior change by individuals (52, 58). Individual endogenous level behaviors that are likely more important than lockdowns may include wearing masks, maintaining distance when encountering individuals (i.e., tailored physical distancing according to risk), and limiting large-size gatherings of individuals (65, 66).

**Table 3:** Peer reviewed published studies suggesting that efficacy of nonpharmaceutical interventions (lockdowns) to prevent spread of COVID-19 are at best highly exaggerated.

**Study**	**Analysis date**	**Numbercountries**	**Details of efficacy of NPI**
Chaudhry et al. (30)	1 April/20	50	A study using data from the top 50 countries ranked by number of cases found that “*rapid border closures, full lockdowns, and wide-spread testing were not associated with COVID-19 mortality per million people*.”
Kuhbandner and Homburg (31)	4 May/20	1	The model in the Nature publication [Flaxman et al. (32)]^a^ used circular reasoning [“the purported effects are pure artifacts”] by “using as an a priori restriction that Rt may only change at those dates where interventions become effective.” In the *UK “the growth factor had already declined*… strongly suggests that the UK lockdown was both superfluous… and ineffective.” In addition, the attribution of the decline in Sweden's Rt to banning of public events is odd because that was an “NPI that they found ineffective in all other countries.”
Islam et al. (33)	30 May/20	149	Implementation of any physical distancing intervention [including lockdown] was *associated with an overall reduction in COVID-19 incidence of only 13%* [IRR 0.87, 95% CI 0.85 to 0.89] in 149 countries. There was no effect on this estimate of days since the first reported case of COVID-19 until the first implementation of physical distancing policies.
Bendavid et al. (34)	Apr/20	10	“After subtracting the epidemic and less restrictive NPI effects [in Sweden and South Korea], we find *no clear, significant beneficial effect of more restrictive NPIs on case growth* in any [of England, France, Germany, Iran, Italy, the Netherlands, Spain, USA] country… The 95% CI excluded 30% declines in all 16 comparisons and 15% declines in 11/16 comparisons.” Plausible “that stay-at-home orders may facilitate transmission if they increase person-to-person contact where transmission is efficient such as closed spaces.”
De Larochelambert et al., (35)	31 Aug/20	160	“Stringencies of the measures settled to fight pandemia, including lockdown, did not appear to be linked with death rate. Countries that already experienced a stagnation or regression of life expectancy [transitioned to older frailer populations], with high income and NCD rates [risk factors including sedentary, poor nutrition, obesity], had the highest price to pay. *This burden was not alleviated by more stringent public decisions*.”
Savaris et al. (36)	26 Aug/20	87 regions	**“**We were not able to explain the variation of deaths/million in different regions in the world by social isolation, herein analyzed as difference in staying at home, compared to baseline… *we found no evidence that the number of deaths/million is reduced by staying at home*.**”**
Leffler et al. (37)	9 May/20	196	In 196 countries by May 9, 2020, *viral testing policies and levels, internal lockdown, and contact-tracing policy were not associated with death rates from COVID-19*.
Gibson (38)	11 May/20	1, with 3,109 counties	“There is *no evidence that [among 3,109 US counties, the less than four-fifths of] counties with a lockdown have fewer deaths* [controlling for 22 confounders].” An example of the “central planning problem: no central planner has all the information (collectively) held by parties involved in voluntary exchange,” and “public health interventions can paradoxically increase infection rates due to risk compensation effects.”
Berry et al. (39)	30 May/20	1 with 50 states	**“***We do not find detectable effects of these [shelter-in-place] policies on disease spread or deaths [among county and states in USA]*.**”**
Homburg (40)	13 Apr/20	9	South Korea had lowest mortality; Sweden had intermediate mortality and did not suffer from ‘exponential growth’; all other countries [Austria, Switzerland, Germany, Spain, Italy, UK, US] had lockdown. **“***All countries that used lockdowns implemented them after the turning points*.”
Gibson (41)	18 Aug/20	34	Across 34 countries, mean stringency index (SI), pre-peak infections SI, and post-peak infections SI were not statistically associated with deaths/M. Pre-peak SI associated at the p <0.10 level, but even then, explains at best 4% of variability in death rates. The SI was not associated with baseline variables, which suggests “that there was a lot of policy mimicry, rather than policy designed to reflect circumstances of each country.”
Krylova (42)	17 Mar/21	1 with 4 states	Mid-sized adjoining Midwest states: Minnesota [hard and extended lockdown] and Wisconsin [short lockdown followed by moderate restrictions]. Minnesota had lower cases (8.9% vs 9.8%), but not lower death rate: *Minnesota 0.12% vs Wisconsin 0.13% [and among those 65+ 0.67% vs 0.66%]*
			Southerly coastal states: California [hard and ongoing lockdown] and Florida [sought every opportunity to ease restrictions and reopen; stay at home rules <1 month]. Florida had slightly higher cases (9.3% vs 9.0%), but not higher death rate: *California 0.15% vs Florida 0.14% [and among those 65+ 0.73% vs 0.55%]*.
Chin et al. (43)	12 Jul/20	14	The model for Europe used in the Nature publication [Flaxman et al. (32)]^a^ was based on circular reasoning [i.e., having modeled Re “as a step function and only allowed to change, immediately so, in response to an intervention”]. Using a model allowing for gradual changes over time and better fitting the data “*lockdown had little or no benefit as it was typically introduced at a point when the time-varying reproduction number was already very low*.” For example, when lockdown was adopted in the UK, Rt had already decreased to 1.11. Overall, “the impacts of lockdown are uncertain and highly model-dependent.”
Gupta et al. (44)	31 July/20	47 states	From pre- to post-reopening of economies, the post-period hospitalization “trend was higher by 1.607 per 100,000 people… nationwide reopenings were associated with 5,319 additional people hospitalized for COVID-19 each day.” But, the “hospitalization rates increased more in states with an active stay-at-home order in place at the time of reopening and in states with phased reopenings,” and “*the change in mortality trend was not significant*.”
Rannan-Eilya et al. (45)	15 June/20	173	Increased time spent at home was associated with increased transmissibility (p=0.15; likely leading to more transmission within households). Reduced time spent in nonresidential locations was associated with no reduction in transmissibility. “Implying that the mobility changes usually associated with lockdowns increased overall transmission globally, although none of these effects was statistically significant.”
Allen (46)	2020	36	Lockdowns have *at best a marginal effect. Ineffectiveness stems from voluntary changes in behavior. Daily deaths per million not negatively correlated with the stringency of lockdown across countries*.
			Several false assumptions in modeling: exogenous behavior [no individual reaction to the virus]; Rt high; IFR high; homogeneous population; missed confounders

*Emphasis added to quotations*.

a *Colombo et al. (47) found that, relaxing the assumption of homogeneity to allow for individual variation in susceptibility or connectivity, a model that has better fit to the data and more accurate 14-day forward prediction of mortality estimated the lives saved were not 3.1 million [as reported by Flaxman et al. (32)], but rather 132.4 thousand, a 23.5-times difference. Lewis discussed several other flaws that make the counterfactual case “completely unrealistic,” and that the inability to explain the Rt decrease in Sweden despite no lockdown questions the accuracy of the modeling (48)*.

**Table 4:** Studies published as preprints suggesting that efficacy of nonpharmaceutical interventions (lockdowns) to prevent spread of COVID-19 are at best highly exaggerated.

**Study**	**Analysis date**	**Numbercountries**	**Details of efficacy of NPI**
Luskin (49)	18 Apri/20	1 with 50 states	Using “highly detailed anonymized cellphone tracking data provided by Google… tabulated by the University of Maryland's Transportation Institute into a ‘social distancing index,”’ it was found that *lockdown severity correlated with a greater spread of the virus*, even when excluding states with the heaviest caseloads, and not with population density, age, ethnicity, prevalence of nursing homes, or general health, suggesting that “[heavy] lockdowns probably didn't help.”
			This analysis also found that states that *subsequently opened-up the most tended to have the lightest caseloads*, suggesting that “opening up [a lot] didn't hurt.”
Atkeson et al. (50)	22 July/20	23 and25 States	An analysis across 23 countries and 25 states each with >1,000 deaths by July 22 found that the growth rates of daily deaths from COVID-19 fell rapidly [from a wide range of initially high levels—doubling every 2–3 days] within the first 30 days after each region reached 25 cumulative deaths, and has hovered around zero or slightly below since.
			Epidemiological models found that this implied both the Re and transmission rates fell rapidly from widely dispersed initial levels [Re≥3], and the Re has hovered around 1 after the first 30 days of the epidemic virtually everywhere in the world.
			The authors suggest that there *must be “an omitted variable bias” accounting for this finding* [and similar findings in previous pandemics], that the role of region-specific NPI's implemented in the early phase of the pandemic is likely overstated, and that the removal of lockdown policies has had little effect on transmission rates.
Wood (51)	27 Jun/20	1	A mathematical model using “a Bayesian inverse problem approach applied to UK data on COVID-19 deaths and the disease duration distribution” suggested that “*infections were in decline before the full UK lockdown* (March 24), and that infections in Sweden started to decline only a day or two later.”
Lundberg and Zeberg (52)	12 Nov/20	25	“The variability in death rates during the influenza seasons of 2015–2019 correlate to excess mortality caused by covid-19 in 2020 (*R*^2^ = 0.48, *p* < 0.0001). In contrast, we found no correlation between such excess mortality and age, population density, degree of urbanization, latitude, GNP, government health spending or rates of influenza vaccinations… *an intrinsic susceptibility to fatal respiratory disease*… was evident long before the arrival of the current pandemic.”
Lally (53)	30 Dec/20	33 and 28	Considering 33 EU countries, controlling for population density and date of first death, the death rate/M up to Dec 30 was *not associated with the maximum or average Stringency Index*. Maximum Stringency Index not predicted by population, GDP/capita, proportion >65 y, household size, nursing home beds/100K, last 2 years flu intensity; suggests “driven by mimicry of neighboring countries.”
			Considering 28 Americas countries, controlling for no land borders with other countries, the death rate/M up to Dec 30 was *not associated with maximum Stringency Index*.
Bjornskov (54)	Jun/20	24	In 24 European countries, comparing by country and week (vs previous 3 years data), lockdown policies are positively associated with mortality development before the mortality rate peaks, have no clear significant relation after the virus has peaked, and therefore “*do not provide evidence suggesting that lockdown policies worked as intended*.”
Kepp and Bjornskov (55)	Nov/20	1 with 11 cities	A quasi-natural experiment in the Danish region of Northern Jutland where 7/11 municipalities in the region went into extreme lockdown while 4/11 retained moderate restrictions. Estimated a non-statistically significant decrease in cases of 2.5% (95% CI −6.3, 1.4%) in locked down municipalities compared to control municipalities. “*We find that an extreme version of societal lockdown had no effect on virus development… may be that lockdown effects have been overestimated*.”
Walach and Hockertz (56)	15 May/20	40	In 40 European and OECD countries, “of the public health variables [closure of borders, schools, or lockdown] only border closure had the potential of preventing cases and none were predictors for preventing deaths. School closures, likely as a proxy for social distancing in severely ill patients [which might be counterproductive in preventing death, as social distance for very ill, and presumably also very old patients, might enhance anxiety and stress] was associated with increased deaths. The pandemic *seems to run its autonomous course and only border closure has the potential to prevent cases*.”
Meunier (57)	24 Apr/20	4	The full lockdown policies of France, Italy, Spain, and UK “haven't had the expected effects on the evolution of the epidemic,” showing “a general decay trend in the growth rates and reproduction numbers 2–3 weeks before the full lockdown policies would be expected to have visible effects,” and “comparison of pre- and post-lockdown observations reveals a counter-intuitive slowdown in the decay of the epidemic after lockdown,” and “*estimates of daily and total deaths numbers using pre-lockdown trends suggest that no lives were saved by this strategy*.”
Wieland (58)	5 May/20	1 with 412 counties	The inflection point [peak] of incident symptomatic cases in Germany occurred at least 3–6 days *before* lockdown (Phase 3), occurred in 62% of counties *before* lockdown, occurred in 12.4% of counties *before* school closures (Phase 2), and in the 3 curfew counties there was no improvement in the curve. “*[R]egional curve flattening seems to have occurred independently from the governmental measures of Phase 2 and 3*. Instead, regional pandemic growth appears as a function of time, reaching the peak of infection rates with a time lag depending on the date the virus emerged.”^a, b^
Agrawal et al. (59)	6 months of 2020 after first COVID-19 death	4350 States and DC	“We *fail to find that shelter-in-place (SIP) policies saved lives*. To the contrary, we find a positive association between SIP policies and excess deaths… countries that implemented SIP policies experienced a decline in excess mortality prior to implementation compared to countries that did not implement SIP policies [the pre-existing trend reversed following implementation of SIP policies]… do not observe differences in excess death trends before and after the implementation of SIP policies based on pre-SIP COVID-19 death rates [the trajectory of the pandemic when policies were implemented]…” Implementation of SIP policies “*does not appear to have met the aim of reducing excess mortality*.”

*Emphasis added to quotations*.

a *Wieland (58) also notes that previous model-based simulation studies [e.g., Flaxman et al. (32)] [as opposed to empirical data such as this] make “a priori assumptions” on the impact of NPI measures, making their results a tautology [i.e., the input intensity of physical contacts between individuals is “set in a way that interventions (such as school closures or social distancing) reduce transmission”]*.

b *In contrast, Dehning et al. (60) reported efficacy of interventions in Germany (March 9 prohibition on large gatherings, March 16 closing of educational institutions and non-essential stores, and March 23 lockdown and contact ban) on reported cases. However, as in Wieland (58), Kuhbandner et al. (61) found that, using data on incident cases, the corresponding growth of infections in Germany reached its maximum on March 5 (long before the first NPI became effective) and was negative since March 16 (i.e., was no longer exponential at time of school closure or extensive lockdown)*.

We acknowledge that some studies have reported efficacy of lockdowns. In the John Snow Memorandum published in Lancet it was claimed that lockdowns were “essential to reduce mortality” (67). The two studies referenced there to support the claim (32, 60) have been refuted by several studies that point out circular and flawed methodology (31, 47, 48, 51), or use of inaccurate data (58, 61) (see Tables 3, 4 for some details of the refuting studies). Other positive studies did not control for the many possible confounders inherent in cross-country comparisons (68). Of interest, several studies have compared the timing of implementation of many different NPIs across countries to determine their (unadjusted for confounders) possible effect on the effective reproductive number (Rt) of SARS-CoV-2 in the respective countries (69–71). Although some NPIs have been suggested as effective using this methodology, these studies have also found the following: substantial variation between world geographical regions in terms of NPI effectiveness (69); less disruptive and costly NPIs can be as effective as more intrusive drastic ones (69); and inconsistent/inconclusive evidence for stay-at-home requirements, public transport closure, international travel controls, testing, contact tracing, and business closures (even finding a paradoxical increase in Rt for some interventions including closure of public transport, stay-at-home requirements, and contact tracing) (69–71). In addition, we question whether the reduction of population-wide Rt is the goal of NPIs; we argued above that the control of hospitalizations and deaths (not simply “cases”) is the goal of NPIs, and Rt may not reflect this for the most vulnerable people in the population.

These findings should not be surprising. Several earlier publications on influenza pandemic management by experts at the Centers for Disease Control, a panel convened by the US Department of Health and Human Services, and the WHO consistently recommended against closing schools, closing large gatherings, quarantine, and border screening, and instead recommended less invasive voluntary measures (72–74). Most recently, in 2019 the WHO recommended against contact tracing and home quarantine of exposed individuals, conditionally recommended workplace closures only as a last step in extraordinarily severe pandemics, and conditionally recommended avoiding crowding for “people who gather in crowded areas (e.g., large meetings, religious pilgrimages, national events and transportation hub locations)” (75). Most governments ignored these previous lessons and written pandemic plans.

## Mistaken Assumption: Lockdowns Have a Favorable Cost-Benefit Balance

In order to deny charter freedoms, “reasonable limits [that are] demonstrably justified” are necessary, which requires the due-diligence of a cost-benefit analysis (76). Several reports find that lockdowns, even if they were to be highly effective, can be predicted to cause *at least* 5–10-times more harm to population wellbeing and deaths in the long-term than they prevent (46, 53, 77–79). Harms include economic recession, unemployment, loneliness, poverty and food insecurity, deterioration of mental health with increased suicides and substance use, increased intimate partner violence and child abuse, lost education and future potential in children, delayed/disrupted health care for serious conditions, and increased societal inequality (79, 80). Framing a recession as being “the economy vs. lives” is a dangerous false dichotomy; as governments can spend less on the social determinants of health, including healthcare, education, roads, sanitation, housing, nutrition, vaccines, safety, social security nets, clean energy, etc., statistical lives will be lost in the years to come (77–79). Importantly, the negative effect of a drop in GDP on population wellbeing and lifespan consistently occur over the long-term, even though not detectable during the short-term due to temporary increased government spending (11, 81–83). Unemployment and loneliness are two of the strongest risk factors for shortened lifespan and chronic diseases (84).

Cost-benefit analyses of each alternative set of possible response measures should be performed, using a common metric that allows making commensurable comparisons among all outcomes, such as the WELLBY or QALY metric (77, 79, 83, 85–87). When done, these analyses have consistently found lockdowns to have higher costs than benefits (Table 5) (46, 53, 77–79, 88–93). Of note, these cost-benefit analyses have made assumptions in favor of lockdowns (i.e., marked reductions in COVID-19 fatalities), and very conservative against lockdowns (e.g., not including the predictable effects of loneliness and unemployment on lifespan and chronic disease, of societal disruption on world food insecurity and poverty rates, of interrupted health care on conditions other than COVID-19; and using the highest estimates of the value of QALY or WELLBY) (11, 77, 84, 92). Therefore, despite the difficulties inherent in complex cost-benefit analyses, the results strongly suggest that lockdowns do not have a favorable cost-benefit balance.

**Table 5:** Cost-benefit analyses of lockdowns (assuming their efficacy) as the response to the pandemic.

**Reference**	**Location considered**	**Benefits considered**	**Costs considered**	**Common metric used for comparison**	**Balance calculation for lockdowns**
Joffe (77)	Global	COVID-19 deaths prevented	Recession (GDP loss), unemployment, loneliness^a^	WELLBY	Minimum 5X higher cost than benefit
Joffe (78)	Canada	COVID-19 deaths prevented	Recession (GDP loss)	WELLBY	Minimum 17X higher cost than benefit
Allen (46)	Canada	COVID-19 deaths prevented	Population wellbeing (assuming people would sacrifice 2-months to have avoided the stringent lockdown)	YLL	Minimum 4.8X higher cost than benefit
Foster (88)	Australia	COVID-19 deaths prevented	Recession (GDP loss attributable to lockdown), wellbeing loss from isolation,^a^ projected suicides, interrupted non-university schooling	QALY	Minimum 6.6X higher cost than benefit
Lally (53)	Australia	COVID-19 deaths prevented; COVID-19 hospitalizations and intensive care admissions prevented; long-COVID in survivors prevented	Recession (GDP loss attributable to lockdown), unemployment^a^	QALY	Minimum 21X higher cost than what is usually considered the benchmark ($100,000 per QALY)
Lally (89)	New Zealand	COVID-19 deaths prevented; COVID-19 hospitalizations and intensive care admissions prevented; long-COVID in survivors prevented	Recession (GDP loss attributable to lockdown)	QALY	Minimum 11X higher cost than a generous benchmark ($146,000 per QALY)
Christakis et al. (90)	USA	COVID-19 deaths prevented	School closures (for median 54 days) induced reduced educational attainment and life expectancy	YLL	98.1% probability that school opening would have been associated with a lower total YLL than school closure
Miles et al. (79)	UK	COVID-19 deaths prevented	Recession (GDP loss)	QALY	Cost per QALY saved far in excess (often by a factor of 10 and more) of that considered acceptable for health treatments in the UK
Rowthorn and Maciejowski (91)	UK	COVID-19 deaths prevented; cost of treatment prevented	Recession (GDP loss)	YLL	Any lockdown is optimal only if 10 YLL is worth £1.68 million, 5.6X higher than official guidelines for drug evaluation (of £300,000)^b^
Ryan (92)	Ireland	COVID-19 deaths prevented	Negative GDP growth, social isolation, surplus unemployment^a^	WELLBY	Minimum 2.5X, and probable 26X higher cost than benefit.
Ekenberg et al. (93)	Romania	COVID-19 deaths prevented	Loss of specific sectors economic activity, recession (GDP loss), loss of human rights, loss of education, loss of mental health, impact on vulnerable groups	Subjective multi-stakeholder rankings of the importance of each aspect and possible response^c^	Mitigation better than Suppression (lockdown) strategy^d^

*GDP, gross domestic product; QALY, quality adjusted life year; WELLBY, wellbeing year; YLL, years of life lost*.

a *The acute effect of loneliness, isolation, or unemployment on experienced wellbeing were considered; however, their strong effects on reducing future lifespan and increasing future non-communicable chronic diseases were not considered*.

b *“To the extent that the government is behaving optimally, these comparisons imply that it values the lives of potential COVID-19 victims a lot more highly than those of other types of victim [p.13]” (91)*.

c *A common metric to allow commensurable comparisons was not used in this study. This resulted in subjective rankings of effects that reflect participants' biases. For example, economic effects were considered much less important than COVID-19 deaths [this assumes the false dichotomy of lives vs. economy], mental health effects and loss of education access were considered much less important than economic effects and COVID-19 deaths [though these factors are known to affect well-being and lifespan], and the impact on vulnerable groups was considered much less important than direct COVID-19 deaths [not appropriate in a population that includes those marginalized groups]*.

d *Mitigation included public communication, encouraging increased hygiene and person protection (stay at home when sick, handwashing, respiratory etiquette, wearing face masks), mild social distancing (large public gatherings banned, work from home where possible, social distancing recommended). Suppression added imposed social distancing measures and restrictions on mobility (school closures, restaurants and large shopping centers closed, and stay-at-home orders)*.

We believe lockdowns reflect the public's unreasonable fear of SARS-CoV-2 and lack of confidence that the government can manage the sick. In order to minimize collateral harms, this fear must be replaced by confidence. Many of the common objections regarding the cost-benefit balance and the necessity of lockdowns are considered in Tables 6, 7 (94–132). Unfortunately, as far as we are aware, governments have not done cost-benefit analyses of the lockdowns that they implemented, and thus have not done their due-diligence that is required by the Charter of Rights and Freedoms in Canada (76, 130).

**Table 6:** Common objections considered regarding the cost-benefit of lockdowns.

**Objection**	**Reply**
The economic recession would happen even without lockdowns, as people still will not work or visit businesses.	This assumes lockdowns are the only option. Using Emergency Management principles to shift from fear to confidence, by protecting people at high-risk of adverse outcomes, ensuring the medical system is robust to manage people with COVID-19, and informing the public their government knows how to deal with the situation, societal disruption and adverse economic impacts can be mitigated.
	These were direct commands to halt work, restrict travel, restrict the number of people inside dwellings, close factory floors, stay at home, etc. At the very least, the recession would have been much less severe without these orders.
	Consensus has been than that the lockdowns are largely responsible for the recession, including by the International Monetary Fund, and the Chief Public Health Officer of Canada (e.g., page 29: “the extensive slowdown in the Canadian economy as a result of public health emergency measures)” (94).
“Long-Haulers” with persistent symptoms will change the cost-benefit balance in favor of lockdowns.	The incidence, severity, and duration of “long-COVID” are not known, and would need to be remarkably high to change the cost-benefit balance in favor of lockdowns. Studies to date do not well quantify the severity and duration of long-term symptoms such as fatigue, breathlessness, “foggy thinking,” etc., making it difficult to interpret the impact (95).
	The highest rates of “long-COVID” are from crowdsourced online data where there is likely a strong participant selection bias. Even app users who had detected COVID-19 cases reported symptoms (of unknown severity) at ≥8 weeks in 4.5% and ≥12 weeks in 2.3%, of whom 43.9% had been hospitalized (96). A recent review suggested ~30% of hospitalized COVID-19 cases have post-acute COVID-19 syndrome (of unclear severity) (97); generously, this would mean that [if <5% of *cases* are hospitalized, then <1% of *infections* are hospitalized] at most 0.3% of infections end up with this syndrome. In the UK Coronavirus Infection Survey the prevalence of any of 12 common symptoms at 12 weeks was 5.0% in SARS-CoV-2 positive patients, and 3.4% in controls, and of continuous symptoms 3.0% in positive patients and 0.5% of controls (98). In children the rates of “long-COVID” approach zero [i.e., similar to background rates of symptoms, especially when correcting the prevalence for the known incidence of multisystem inflammatory syndrome in children, which occurs in <1/3,100 infections (99)] (98–103).
	Most reports do not compare to contemporary controls during the pandemic, controls who are often experiencing social isolation, unemployment, loneliness, and ~30% prevalence of anxiety and depression (104, 105).
	Nocebo effects due to being bombarded by reports in the press and on social media, anxiety, fear, and negative expectations can lead to at least some of the cases (106).
Healthcare capacity can be predicted to be overwhelmed without lockdowns.	Forecasting of healthcare capacity needs in the short or medium term, even when built directly on data and for next day predictions, has consistently failed (107), and most healthcare systems were not overwhelmed [including in Sweden (108)] despite sometimes being stressed with high peaks of cases (1).
	This assumes both that lockdowns are the only way to preserve capacity, and that we cannot develop surge capacity. A better, focused, far less harmful option using Emergency Management principles to protect those people at high-risk, and ensure surge capacity (without shutting down other healthcare), is more likely to prevent the healthcare system from being overwhelmed.
	This assumes preserving healthcare capacity is the only goal; however, preventing the most harm to society *as a whole* is the goal. The effect of overwhelmed healthcare capacity would need to be remarkably high to change the cost-benefit balance to be in favor of lockdowns.
	This assumes we cannot maintain the healthcare workforce. However, much of healthcare workforce depletion has been “self-inflicted”: healthcare workers should be allowed to work if asymptomatic and universal masking is in place (109); avoiding school closure can prevent over 15% of healthcare workforce losses (due to childcare responsibilities) (110); well organized voluntary re-deployment of retired staff and rapid on-the-job training of senior students can contribute to maintaining the healthcare workforce.
The variants “of concern” (VOC) are more transmissible and deadly.	That VOC are more transmissible is based on mathematical modeling, and not certain (111). Another mathematical model showed that the course of the UK pandemic was not altered by the emergence of the B.1.1.7 VOC (112).
	That VOC are more deadly is based on studies that used the same UK data, excluded >50% [and even more of the deaths] of the population from analyses for “missingness,” included only 8% of UK COVID-19 deaths, did not control for co-morbidities as a confounder, and only examined *case* fatality rate (113, 114). It may be more likely the surge in deaths in the UK “was generated by pandemic fatigue and not the new variant of the virus” (115). Another study found no increase in case-fatality-rate from the UK VOC (116). The recent surge in India with the delta VOC still (as of April 30, 2021) had India ranked in cases and deaths per capita at 114^th^ and 116^th^ in the world, and (on April 30, 2021) with daily deaths per million (1.95) and total deaths per million (151) lower than in the European Union (4.68 and 1,548, respectively) and worldwide (1.65 and 408 respectively) (117).
	If accurate, a 30–60% increase in *case* fatality rate would mean Emergency Management principles are even more important to protect the older population, as the *infection* fatality rate would still be very low in younger people [since there would be no difference in relative risk increase by age (111, 112, 114)], and the cost-benefit balance still very strongly favor no lockdowns.
	Current vaccines are effective for the UK B.1.1.7 VOC. Vaccines may be less effective for the South African B.1.351 and Delta VOC; however, emerging data suggest high efficacy for preventing severe COVID-19, hospitalization, and death (118), and this efficacy likely remains at least due to T-cell immune mechanisms (119, 120).

**Table 7:** Common objections to the alternative response using emergency management principles.

**Objection**	**Reply**
It seems as if every country has used lockdowns. How could so many be wrong?	*Groupthink:* the tendency for groups to let the desire for harmony and conformity prevail, resulting in dysfunctional decision-making processes and being less willing to alter course of action once settled upon (77, 121). - NPIs spread to ~80% of OECD countries within a 2-week period in March 2020. A main predictor of a country implementing NPIs was prior adoptions of a policy among spatially proximate countries, i.e., the number of earlier adopters in the same region. Variables not predicting adoption of NPIs included the number of cases or deaths, population >65 years old, or hospital beds/capita in the country (122).
	*Cognitive biases:* the triumph of deeply human instincts over optimal policy (77). - identifiable lives bias: we ignore hidden “statistical” lives - present bias: we prefer immediate benefits to even larger benefits in the future - availability bias: the vividness of COVID-19 deaths captured attention - anchoring bias: we disregard evidence that disproves our favorite theory - escalation of commitment bias: we invest more and more resources into a set course of action (121) - loss aversion: we avoid realizing the losses from a course of action (121)
	*Crowd effects:* a type of contagious mass hysteria due to escalation of fear, anxiety, and panic perpetuated by popular and social media (106). - we became united in crowds, acting together against a common threat, in a war against an invisible enemy that *will be* won, with a “disregard and disinterest on the part of individuals in the enormity of the collateral damage, either to their own kids, people in other countries, their own futures…” (123). - the “parasite hypothesis” is supported by evidence that “subjective perception of infection risk causes individuals to be more conformist, to prefer conformity and obedience in others, to respond more negatively toward others who fail to conform…” (124).
The goal should be COVID-zero. Australia, Japan, New Zealand, South Korea, Taiwan, and Singapore have low death rates and have opened their society.	This assumes that the low rates were causally due to quick harsh prolonged lockdowns that suppressed transmission to zero. But these countries had lower (and variable) severity of lockdowns than most other countries. The “success” was most likely because they are islands that could strictly close their international borders (not having essential land-based supply-chains) (125). In Canada for example, there are over 20,000 trucks a day that supply our essentials (especially for our food system) from the United States, making strict border closure impossible (126).
	This assumes the cost-benefit balance favors prolonged lockdowns. But this is not the case, and even analyses in Australia and New Zealand find the balance strongly against lockdowns (53, 89).
	This assumes an exit strategy from COVID-zero. But these isolated countries find themselves in a world where SARS-CoV-2 is endemic, with unpredictable ongoing threat of breakthrough cases and sudden lockdowns (126). Since vaccine immunity is not lifelong, not adept at preventing secondary transmission or reinfection with some viral variants, and with limited vaccine supplies and poor vaccine uptake, population vaccination does not seem to be an achievable COVID-Zero exit strategy (127). In Australia for example, at the end of June 2021 another stay-at-home order (a “snap lockdown)” was issued for small clusters of cases (27 cases/day), and is being extended to July 16 for >5 million people (128). In Israel vaccine has controlled healthcare burden and deaths, but transmission of the delta variant continues, and the country requires no lockdown (129).

## Mistaken Assumption: Lockdowns are the Only Effective Option

The pandemic is better framed as a public emergency, not a public health emergency. This is because the pandemic is affecting all sectors of society (not just the healthcare system), and the process of emergency management (EM) is necessary to respond. A public emergency response aims to minimize the impact of the hazard, SARS-CoV-2, on our *society as a whole* (130–132). This requires a written EM plan released to all citizens. A coordinating agency [in Canada, the provincial EM Agency] is required to coordinate requests from the Subject Matter Agency [public health] dealing with the direct effects of the virus, while also dealing with the indirect effects of the pandemic.

Emergency Management (EM) is the prevention and mitigation of, preparedness for, response to, and recovery from emergencies regardless of the hazard/risk (130, 132). Emergency Management Agencies (EMA) are charged with this mitigation, preparedness, response, and recovery to all hazards (130, 132). These EMA are established, staffed, trained, and equipped to manage the governance, operations, planning, intelligence, logistics, communications, finances, administration, public/private sector collaboration, and training necessary to respond to any emergency (132). There are defined steps in this process. First is identification of the hazard, in this case SARS-CoV-2. Second is selection and maintenance of the aim (or mission), in this case to minimize the impact of SARS-CoV-2 on the jurisdiction (i.e., not to flatten the curve, or protect the medical system, which are incorporated into objectives). Third is to establish a comprehensive Governance Task Force to provide leadership for all policy, programs, and actions taken, led by the Premier. Fourth is a risk/hazard assessment to give detailed assessment of the risk (e.g., the extremely age-dependent mortality especially with comorbidities, impacts on critical infrastructure including healthcare, specific considerations for socially vulnerable groups). Fifth is the Mission Analysis that lays out a list of objectives of *what* needs to be done, including tasks given (pre-written) and tasks implied, required to meet the Aim. This always includes ways to maintain confidence in government and diminish fear, ensure mutual aid, and ensure constant communications, and in this case would also include objectives to protect seniors, and to protect critical infrastructure and essential services (e.g., new medical surge capacity, full continued education, continuity of business and economy). Sixth is Defining Courses Open/Options, *how* the objectives can be met, a series of courses open for each grouping of tasks as determined by assigned teams with appropriate diverse expertise (to prevent groupthink). Each course open has a full assessment of advantages/disadvantages (i.e., a cost-benefit analysis) to justify options and plan for solutions to collateral damage. Seventh is public issuing of a written, comprehensive, evidence-based pandemic response plan, which forms the basis of confidence in government, is a statement of transparent demonstrably justified due diligence, and establishes that the government has a plan, is ready to respond, and is open to suggestions to improve the plan. This giving citizens of voice is important for trust in government and policy acceptance (121).

In the 2014 Alberta Pandemic Response plans the four goals were: controlling the spread of infection and reducing illness and death from the virus, mitigating societal disruption by ensuring continuity and recovery of critical services, minimizing adverse economic impact, and supporting an efficient and effective use of resources (133). These form the basis of the Mission Analysis, overarching objectives (tasks given) in the EM process, and are broken down into smaller manageable tasks; for example, care of the most at risk, assurance of medical capacity, assurance of education, etc. Similarly, the four concurrent critical functions of EM are mitigation (i.e., attempt to separate the threat from the potential targets, or separate the targets from the potential threat), preparedness (e.g., building capability to effectively and rapidly respond when items at risk are affected by the hazard), response (e.g., execution of the capability to prevent injury and loss of life, protect property and critical resources, and meet basic human needs), and recovery (e.g., re-establishment of the economy and a state of normal life) (131, 132). These functions guide the Mission Analysis and Courses Open/Options steps of the process.

Others have suggested that decision-making has been made under “suboptimal conditions” of high stakes, time pressure, complexity, and uncertainty, leading to information-processing failures and poor outcomes (121). Further, they suggest “using reflexivity to counteract” these failures (121). The information-processing failures included several steps. First, a failure to search for and share relevant information, especially due to groupthink [e.g., “a biased sampling of information,” a “focus on agreement at all costs,” including ignoring or suppressing information not in line with the majority view] (121). Second, a failure to elaborate on and analyze information, especially due to framing effects [e.g., framed narrowly as the number of lives *lost*, only from COVID-19; as an action-oriented “war against an invisible enemy” with “warriors” on the “frontline” and “traitors” questioning the response] (80, 121). Third, a failure to update conclusions in the light of new information, especially due to escalation of commitment [“investing more resources in a set course of action, even in the face of clear evidence that it is not working;” giving clarity of direction by sticking to a chosen course of action] (121). Team reflexivity, “a deliberate process of discussing team goals, processes, or outcomes—can function as an antidote to biases and errors in group decision-making,” taking “steps to maximize the decision-making process and increase the chances of positive outcomes” (121). This involves several steps. First, a holistic approach, that focuses on “widening the array of opinions considered” and “avoiding an overreliance on experts” (121). Second, a frame that is broader (i.e., “societal well-being”), that “explicitly considers and weights possible consequences for a variety of societal stakeholders,” while “increasing the number of options or solutions considered” (121). Third, an ongoing process, one that constantly reassesses the situation and is “willing and able to reflect on the actions they have taken, and, when necessary, are prepared to change the current direction or make adjustments [based on the evidence]” (121). Overall, there is accountability for the decision-making process. This is what the Emergency Management process is all about: requiring that all stakeholders are included in a defined process that demands all tasks, given and implied, are developed with options based on detailed cost-benefit analysis and continual feedback so that leadership can make fully informed decisions.

We believe that these critical functions were not achieved by the lockdown approach, with ongoing deaths in long-term care homes and seniors (poor mitigation), societal disruption and economic devastation (poor recovery and response), and sub-optimal use of resources (poor preparedness and response). The concept of precision shielding can demonstrate the failure of lockdowns in many countries, including Canada. Ioannidis proposed the shielding ratio, “defined as the ratio of prevalence of infection among people in a high-risk group vs. among people in a low-risk group” that can be estimated as follows: (G) (IFR_l_) (1-f_h_)/(IFR_h_) [(fh-(G)(f_h_)], where G is the proportion of COVID-19 deaths contributed by the high-risk group, f is the relative share of the high-risk (h) or low-risk (l) group to the general population, and IFR is the infection fatality rate in the high-risk (h) or low-risk (l) group (134). For the elderly high-risk group (age ≥70 years) Canada had a shielding ratio very close to 1.0 (i.e., no shielding), and Spain and China had inverse shielding (the elderly were infected more frequently than younger populations) (134). For the nursing home residents high-risk group, inverse shielding occurred in Belgium, UK, Spain, and likely USA (134). Performing the calculation for nursing home residents in Canada as (0.73) (0.002) (1–f_h_)/(0.25) [(f_h_-(0.73) (f_h_)] with f_h_ being 160,000/36,110,000, the shielding ratio was 4.9 (i.e., strong inverse shielding). In addition, for example in the United States, minority groups have had higher rates of age-adjusted hospitalizations and deaths by a factor of 2.9 and 2.0 for African Americans, 2.8 and 2.3 for Hispanic or Latino, and 3.3 and 2.4 for American Indian or Alaska Native groups, again inverse shielding (14, 135). This suggests that the non-focused measures used (i.e., lockdowns) did not protect the most vulnerable in Canada or elsewhere, leaving nursing home personnel and residents more exposed than the rest of the population, and exacerbating social inequalities in the population (134). Despite the adverse and unequal effects of lockdowns on the general population (e.g., loneliness, mental health deterioration, unemployment, recession, interrupted healthcare, and interrupted education), and the attempts to provide surge “capacity” in healthcare (e.g., by interrupting healthcare for non-COVID-19 illness), the most vulnerable groups were not protected and were even put at higher risk.

Many others have made the point that less restrictive mandates for social distancing should have been considered; this includes the studies discussed above that found more stringent measures were not associated with reduced viral transmission and deaths, and the cost-benefit studies finding stringent lockdown measures to have much higher costs than benefits. Many diverse stakeholders should be involved in planning to ensure that all costs and benefits, and all possible alternative measures are considered in a deliberative process that aims to determine the best response while minimizing cognitive biases (77, 122). Using EM principles, better priorities are clear.

## Discussion: Priorities to Achieve Better Outcomes

First, we must protect those most at risk by separating them from the threat (mitigation). This means protection of concentrations of older people, particularly in long-term care homes. Residents and staff should be placed in quarantine, with volunteer staff asked to work 1-month-on and 1-month-off living away from their families with generous compensation. This may be done in new larger facilities to reduce staffing demands. More well-paid, well-trained staff working in only one facility with adequate personal protective equipment are required. This can prevent most deaths and much of the challenges to hospital capacity. Other seniors with multiple severe co-morbidities also require voluntary quarantine, with plans for either separate housing in temporary long-term-care-like facilities, or in-home quarantine with a voluntary primary and secondary caregiver, and home delivery of groceries and other essentials (63, 64). This would prevent most other deaths and challenges to hospital capacity. In effect, focused protection “reduces COVID-19 mortality by better protecting the elderly and other high-risk groups [while] children and low-risk adults, for whom lockdowns cause more physical and mental suffering than COVID risk does, are encouraged [for those who decide] to live near-normal lives” (63, 64, 136). This is based on evidence of the extreme age-dependent risk from COVID-19, and the extreme harms of population-wide lockdowns.

Second, we must ensure critical infrastructure is ready for people who get sick, by being capable (prepared) and executing this capability (response) to protect and treat those affected by the threat. This means building *new* surge capacity in hospitals, *without* canceling or delaying healthcare for diseases other than COVID-19. Public health leaders should be tasked with ensuring we build and segregate an appropriate number of intensive-care and hospital beds to handle any surge. Healthcare workers who are asymptomatic (and exposed or even swab positive) can continue to work in hospitals with universal staff masking policies as transmission is rare (109). The healthcare workforce can also be better preserved by not closing schools [which can deplete over 15% of the workforce due to childcare responsibilities (110)], voluntary re-deployment of retired staff, and rapid on-the-job training of senior nursing and medical trainees.

Third, we must shift the focus of response from fear to confidence. Do not chase case counts, as reducing hospitalization and mortality are the goals of mitigation. Do not lockdown everybody, as this causes immense economic and collateral damage. Keep schools open, as this prevents the predictable adverse effects on children from loss of socialization, education, earning potential, and future lifespan. In addition, children are at lower risk from SARS-CoV-2 than from seasonal influenza (Table 1), school transmission is rare, and teachers have similar or lower risk of SARS-CoV-2 transmission than other workers of their age (137–141). Removing fear will be very difficult. The public has been bombarded with fear-inducing information from the media (80, 121). Government intentionally (e.g., in the UK) or unintentionally encouraged covert psychological strategies to induce compliance with lockdowns (142, 143). This has been done by inducing fear (an inflated perceived threat level), shame (conflating compliance with virtue), and peer pressure (portraying non-compliers as a deviant minority) (142, 143). This included presenting daily death and case counts without context (i.e., without denominators, background death rates, or information about the extreme age-dependent risk), inflated predictions of future cases and deaths (using flawed forecasting) (107), repeated pictures from worst case examples of how terrible the illness can be (implying how deadly the virus can be to *everyone*), warning that hospital capacity will be overwhelmed if there is noncompliance, and using scary slogans (e.g., telling children not to kill their grandparents, and that normality is only possible if they get vaccinated). This may have involved “agnotology”, “the ways ignorance or doubt about certain topics is [culturally] created by means of withholding or presenting information in a certain way” (80). For example, framing as loss (i.e., deaths) and “war” with censorship of dissent (e.g., erosion of civility in academic discourse) (144–146), and reliance on social influence (e.g., group conformity, and obedience to authority) and superstitious bias (e.g., a need to see a relation between behavior, particularly sacrifices, and outcomes, even if this relation is not there) (80).

The fear can only be shifted to confidence using honest clear information provision and rational argument (147, 148). Daily messaging and presentations delivered personally by government leaders must repeatedly focus on educating the public on the difficult trade-offs involved (i.e., the benefits and harms of lockdowns, including their massive collateral damage and the false dichotomy of lives vs. the economy), the risks from SARS-CoV-2 (i.e., the extreme age-dependent risk of severe outcomes, particularly in people with co-morbidities) placed in context of other diseases and causes of death (risks the public routinely face) (147, 148), and the justification for the priorities of focused protection of people at high-risk of adverse outcomes (including how this will be done) and ensuring the medical system is robust to manage people with COVID-19 (including plans for surge capacity). By informing the public in this way that its government knows how to deal with the situation and has a written pandemic plan, the fear, societal disruption and adverse economic impacts can be mitigated. This shift will be gradual and require repeated clear accurate messaging. It will require admitting errors, explaining that there will be deaths and suffering whatever is decided, and that the goal is to have the least possible death and suffering. In addition, many have experienced emotions similar to grief, with emptiness, sadness, and loss of meaning in life from a loss of normalcy, and functional coping strategies have been blocked by lockdowns (e.g., eating healthily, seeking social support, and exercise). Improving access to better coping strategies will be important (80, 149, 150).

Each of these priorities must also include a strong health equity perspective, attempting to address upstream/midstream policy and institutional factors, and to implement downstream rapid responses in ways that ensure the well-being of all of society, including socially vulnerable groups (11, 13, 14, 81). The aim of the EM process is to design a system to protect the most at risk, wherever they are found, regardless of race, color, religion, socioeconomic level, sexual orientation, etc. Critical steps to operationalize in order to fulfill the four EM critical functions, and achieve these three priorities, are shown in Table 8, with equity considerations included.

**Table 8:** Next steps for a better way forward focusing on the concurrent emergency management functions.

**Emergency management function (132)**	**Step to be taken^a^**	**Some health equity considerations^b^**
Preparation	Release a comprehensive written Pandemic Response Plan, showing what is to be done by phase, triggers for moving between phases, and what the public's role is in each phase. Define the mission: to ensure minimum impact of SARS-CoV-2 on society *as a whole*. Be open to public suggestions to improve the plan.	Aim to improve equitable access to material conditions for health: food security; housing security; health care insurance coverage; sufficient community health centers and health care providers; prohibit evictions, rent hikes, and water and utility shutoffs during the crisis.
Mitigation	Vigorously enact a plan to protect our most vulnerable. Have a separate plan for long-term care homes, and for care of those ≥60 years with multiple co-morbidities not in long-term care homes. Produce risk analysis for population so family physicians can give advice to their patients based on age and comorbidities.	Aim for plans to include socially vulnerable groups, for example, to reduce household crowding with temporary housing support, prioritize economic relief, improve infection prevention and control support in workplaces, and provide voluntary alternate housing for those at highest risk.
Response	Ensure all critical infrastructure (including but not limited to hospitals) is ready for people who get sick and who need to take sick days. New surge capacity in hospitals is required such that continuity of our medical system is ensured. Evidence on existing and surge capacity and the mutual aid available will need to be shared constantly.	Aim for equitable access to quality healthcare, for example, mobile units, extended hours, free transportation, suspended requirements for insurance and documentation of residence, follow-up care at no cost.
	Continue to vaccinate as vaccines become available, for the current strains of SARS-CoV-2. Target those at highest risk, to ensure a favorable risk-benefit balance for the individual.	Aim for equity of access: partner with trusted community sources to promote awareness and uptake; manage transportation barriers, simplify registration procedures.
Recovery	Remove the fear campaign from the media (without press control). This needs a plan and will not be easy. Government daily information must be repeatedly presented with context of total hospital capacity, plans for surge capacity, other diseases and risks causing death annually compared with COVID-19 death rates (i.e., with denominators) by age group. Explain what the difficult trade-offs are and justify why focused protection is a better response. Issue a written pandemic response plan to show the public there is a plan and their government is ready, knows how to deal with the situation, and is protecting the province while minimizing restrictions on civil liberties.	Aim for improved communication by engaging trusted community organizations and leaders for messaging that is at appropriate reading level, in multiple languages, and viewed as credible.
	End talk of future lockdowns and loosen social distancing rules. Evidence on the cost-benefit balance of lockdowns will need to be shared constantly.	Aim to not criminalize vulnerability where social distancing and working from home are not possible.
	Guarantee to keep schools and day cares open, with relaxed social distancing, regardless of whether children are vaccinated. Evidence on the risk posed to children and teachers by age group will need to be shared constantly.	Aim that education be available to all, including those with the fewest opportunities (to avoid worsening social disparities that education systems are intended to level).
	Get everyone <65 years without comorbidities who can and want to work fully back to work.	Aim to improve basic economic security: living wage with paid sick leave; easy access to unemployment benefits and public assistance if needed.

a *These steps overlap emergency management functions, but for simplicity we assign each to the most important function the step addresses. These also address the four goals in the 2014 Alberta Pandemic Response plans [page 9 (133)]. Note that the four emergency management functions should occur concurrently, not sequentially*.

b *See references (11–13, 151) for more discussion of equity considerations*.

## Conclusion: There is a Better Way Forward

Based on EM principles, the age-dependent risk from SARS-CoV-2, the minimal (at best) efficacy of lockdowns, and the terrible cost-benefit trade-offs of lockdowns, we suggest that an EM Agency (e.g., in Canada, in each province) take charge of coordinating the pandemic response. We need to reset the pandemic response so that we can manage risk and save more lives from both COVID-19 and lockdowns, thus achieving far better outcomes in both the short- and long-term.

## Author Contributions

AJ and DR contributed to conception and design of the work, acquisition, analysis and interpretation of the data, and substantial critical revisions of the manuscript for important intellectual content, have approved the submitted version, and have participated sufficiently in the work to take public responsibility for the content. AJ wrote the first draft of the article. All authors contributed to the article and approved the submitted version.

## Conflict of Interest

The authors declare that the research was conducted in the absence of any commercial or financial relationships that could be construed as a potential conflict of interest.

## Publisher's Note

All claims expressed in this article are solely those of the authors and do not necessarily represent those of their affiliated organizations, or those of the publisher, the editors and the reviewers. Any product that may be evaluated in this article, or claim that may be made by its manufacturer, is not guaranteed or endorsed by the publisher.

## Abbreviations

COVID-19: Coronavirus Disease 2019
EM: emergency management
IFR: infection fatality rate
SARS-CoV-2: Severe Acute Respiratory Syndrome Coronavirus-2.
